# Unveiling the Nature and Strength of Selenium-Centered Chalcogen Bonds in Binary Complexes of SeO_2_ with Oxygen-/Sulfur-Containing Lewis Bases: Insights from Theoretical Calculations

**DOI:** 10.3390/ijms25115609

**Published:** 2024-05-21

**Authors:** Tao Lu, Renhua Chen, Qingyu Liu, Yeshuang Zhong, Fengying Lei, Zhu Zeng

**Affiliations:** School of Basic Medical Sciences/School of Biology and Engineering, Guizhou Medical University, Guiyang 550025, China; lutao0409@gmc.edu.cn (T.L.); chenrenhua5288@163.com (R.C.); l.qingyu@foxmail.com (Q.L.); zhongyeshuang@foxmail.com (Y.Z.)

**Keywords:** quantum chemical calculations, binary clusters, chalcogen bonds, π–hole interactions

## Abstract

Among various non-covalent interactions, selenium-centered chalcogen bonds (SeChBs) have garnered considerable attention in recent years as a result of their important contributions to crystal engineering, organocatalysis, molecular recognition, materials science, and biological systems. Herein, we systematically investigated π–hole-type Se**∙∙∙**O/S ChBs in the binary complexes of SeO_2_ with a series of O-/S-containing Lewis bases by means of high-level ab initio computations. The results demonstrate that there exists an attractive interaction between the Se atom of SeO_2_ and the O/S atom of Lewis bases. The interaction energies computed at the MP2/aug-cc-pVTZ level range from −4.68 kcal/mol to −10.83 kcal/mol for the Se**∙∙∙**O chalcogen-bonded complexes and vary between −3.53 kcal/mol and −13.77 kcal/mol for the Se**∙∙∙**S chalcogen-bonded complexes. The Se**∙∙∙**O/S ChBs exhibit a relatively short binding distance in comparison to the sum of the van der Waals radii of two chalcogen atoms. The Se**∙∙∙**O/S ChBs in all of the studied complexes show significant strength and a closed-shell nature, with a partially covalent character in most cases. Furthermore, the strength of these Se**∙∙∙**O/S ChBs generally surpasses that of the C/O–H**∙∙∙**O hydrogen bonds within the same complex. It should be noted that additional C/O–H**∙∙∙**O interactions have a large effect on the geometric structures and strength of Se**∙∙∙**O**/**S ChBs. Two subunits are connected together mainly via the orbital interaction between the lone pair of O/S atoms in the Lewis bases and the BD*(OSe) anti-bonding orbital of SeO_2_, except for the SeO_2_**∙∙∙**HCSOH complex. The electrostatic component emerges as the largest attractive contributor for stabilizing the examined complexes, with significant contributions from induction and dispersion components as well.

## 1. Introduction

Selenium (Se) is the third stable element in group VI of the periodic table, and its abundance in the Earth’s crust is about 5 × 10^−6^%. Selenium is an important nutrient for humans and other animals, playing a crucial role in various physiological functions [1,2], and its deficiency in living organisms can cause various serious diseases [2]. Despite its importance, selenium is also a toxic element for humans even in small doses, which can cause selenosis [2]. Selenium exists in a variety of inorganic and organic selenium-containing compounds, including selenocysteine and selenomethionine found in biological molecules [3]. These selenium-containing compounds show great potential applications in medicinal chemistry [3], organic synthesis [4,5], materials science [6,7], and biochemistry [8,9]. It has been observed that the selenium in these selenium-containing compounds can engage in different non-covalent interactions (NCIs) with partner molecules, in analogy to the behavior of sulfur. These NCIs are crucial for the stability, structure, and function of compounds and proteins containing selenium, as well as for molecular recognition [10,11,12,13,14,15,16,17]. Theoretical and experimental studies have demonstrated that selenium can serve as a proton acceptor to engage in D–H**∙∙∙**Se (D = C, N, O, S) hydrogen bonds (HBs) [18,19,20,21,22,23] and also act as a proton donor to form Se–H**∙∙∙**A (A = N, O, S, Se, π) HBs [24,25,26,27,28]. These selenium-centered hydrogen bonds (SeCHBs) are a significant class of selenium-containing NCIs.

In addition to SeCHBs, selenium also exhibits the ability to form chalcogen bonds (ChBs) with electron-rich sites. Chalcogen bonds have been defined as the attractive NCIs between an electrophilic region related to a covalent-bonded group VI atom (O, S, Se, Te) and a nucleophilic region within the same or another molecular entity [29]. In recent years, ChBs have garnered considerable attention from theoreticians and experimentalists owing to their important contributions to crystal engineering [30,31], molecular recognition [17,32], organocatalysis [33,34], drug design [6,35], materials science [36,37], and biological systems [13,38,39]. ChBs belong not only to a subclass of σ–hole interactions but also to a subclass of π–hole interactions [40,41]. The former pertains to the σ–hole region of a chalcogen atom situated on the extension of the R-Ch covalent bond (where Ch denotes a chalcogen atom), and the latter is concerned with the π–hole region of a chalcogen atom positioned orthogonal to the planar skeleton of the molecular entity [40,41]. Chalcogen atoms can act as electron acceptors (ChB donors), electron donors (ChB acceptors), or fulfill both roles in ChBs [15]. It has been found that ChBs are similar to HBs [42] and, in certain instances, even stronger than HBs in terms of strength, especially for chalcogen atoms with larger radii. The strength of these ChBs depends on both the chalcogen atom involved in the bonding and the groups covalently bonded to it [15,43,44]. Concretely, a ChB’s strength increases with increases in the radius of the electron-poor chalcogen atom, but gradually decreases as the radius of the electron-rich chalcogen atom grows [45]. Furthermore, the existence of an electron-withdrawing group covalently attached to the electron-deficient chalcogen atom and an electron-donating group connected to the Lewis base can enhance the ChB strength. Additionally, it is also reported that the attractive nature of these ChBs is mainly determined by the dominant contributions of electrostatic, dispersion, and charge-transfer components [46].

Currently, quantum chemical calculations and several spectroscopic techniques such as X-ray diffraction and NMR spectroscopy have been mainly used to investigate selenium-centered chalcogen bonds (SeChBs). These SeChBs mainly include Se**∙∙∙**N [47,48,49,50,51,52], Se**∙∙∙**O [10,13,47,51,53,54], Se**∙∙∙**S [47,55], Se**∙∙∙**Se [28,56,57], and Se**∙∙∙**π [58,59,60] contacts. Zhang [48], Mugesh [61], and Panda [11] and colleagues theoretically investigated the impacts of the substituents covalently bonded to the Se atom, hybridization of the nitrogen atom, the chelate ring, and rigidity on Se**∙∙∙**N ChBs. Wang and coworkers [51] performed an X-ray crystallographic study and disclosed that intermolecular and extremely strong intramolecular Se**∙∙∙**N/O ChBs play a dominant role in stabilizing the crystal structures of the polymorphs of *ortho*- and *para*-nitrophenyl selenocyanate. Carugo and coworkers [13] conducted a statistical analysis of crystal structures obtained from the Protein Data Bank (PDB) and revealed that there exists a significant abundance of Se**∙∙∙**O ChBs, which play a pivotal role in the stability, structure, and function of proteins and peptides. Thomas et al. [53] observed a very short Se**∙∙∙**O ChB with a binding distance of 2.522 Å in the polymorphs of the organoselenium antioxidant ebselen. Bauzá and colleagues [55] also demonstrated the significance of intermolecular Se**∙∙∙**O/S ChBs in the stabilization of protein–ligand complexes involving Se–pyranose through PDB surveys. Veljković et al. [57] conducted a statistical analysis of crystallographic data from the Cambridge Structural Database (CSD), revealing the cooperative roles of Se**∙∙∙**Se ChBs and C/Se–H**∙∙∙**Se HBs in stabilizing crystal structures of organoselenium compounds. Tskhovrebov and coworkers [59] reported that symmetrical dimers of benzylic-substituted 1,2,4-selenodiazolium salts are stabilized through Se**∙∙∙**N and Se**∙∙∙**π ChBs, where the former are weaker than the latter. It is important to acknowledge that the above-mentioned SeChBs mainly belong to the group of σ–hole interactions, with a focus on the divalent selenium atom in the existing literature. However, studies on the π–hole interactions of the hypervalent selenium atom as the ChB donor remain limited to date [45,62].

Similar to sulfur, selenium can also participate in tetravalent bonding in compounds such as SeO_2_, which serves as a ChB donor. SeO_2_ is a good prototypical molecule for studying π–hole-type ChBs, as it can interact with various Lewis bases. For instance, Esrafili and coworkers [62] computationally investigated the cooperativity between π–hole-type Se**∙∙∙**N ChBs and σ–hole-type halogen bonds in CH_3_**∙∙∙**XCN**∙∙∙**SeO_2_ complexes (X = F, Cl, Br, and I). Recently, we conducted a systematic examination of the π–hole-type chalcogen**∙∙∙**chalcogen interactions in XO_2_**∙∙∙**CH_3_YCH_3_ complexes (X = S, Se, Te; Y = O, S, Se, Te) [45]. The results indicated that these X**∙∙∙**Y ChBs are strong in strength and have some degree of covalent character. Energy decomposition analysis data revealed that the electrostatic component provides the largest contribution to the stabilization of these complexes, while the dispersive component is comparable to the induction component in terms of its contribution. It is well known that oxygen and sulfur, as the first two stable elements of group VI, are ubiquitous in various inorganic and organic compounds, including CH_3_OCH_3_ and CH_3_SCH_3_. These oxygen- and sulfur-containing compounds can serve as different types of Lewis bases and participate in the formation of a range of σ– and π–hole interactions. Herein, we systematically studied the π–hole-type Se**∙∙∙**O/S ChBs in binary complexes of SeO_2_ with a series of O-/S-containing Lewis bases employing high-level ab initio computations. Simultaneously, the strength and nature of such Se**∙∙∙**O/S ChBs were comprehensively evaluated and characterized by means of the molecular electrostatic potential (MEP), quantum theory of atoms in molecules (QTAIM), non-covalent interaction plot (NCIplot), natural bond orbital (NBO), and symmetry-adapted perturbation theory (SAPT) methods. Additionally, it has been shown that the substitution and hybridization effects of nitrogen bases significantly affect the strength of σ–hole-type Se**∙∙∙**N chalcogen bonds [49]. Therefore, we also explored the effects of electron-donating methyl and hydroxyl groups and additional hydrogen bonds on the geometric structures and strength of the π–hole-type Se**∙∙∙**O/S ChBs in this study.

## 2. Results and Discussion

### 2.1. MEP Analysis of Monomers

**Figure 1:**
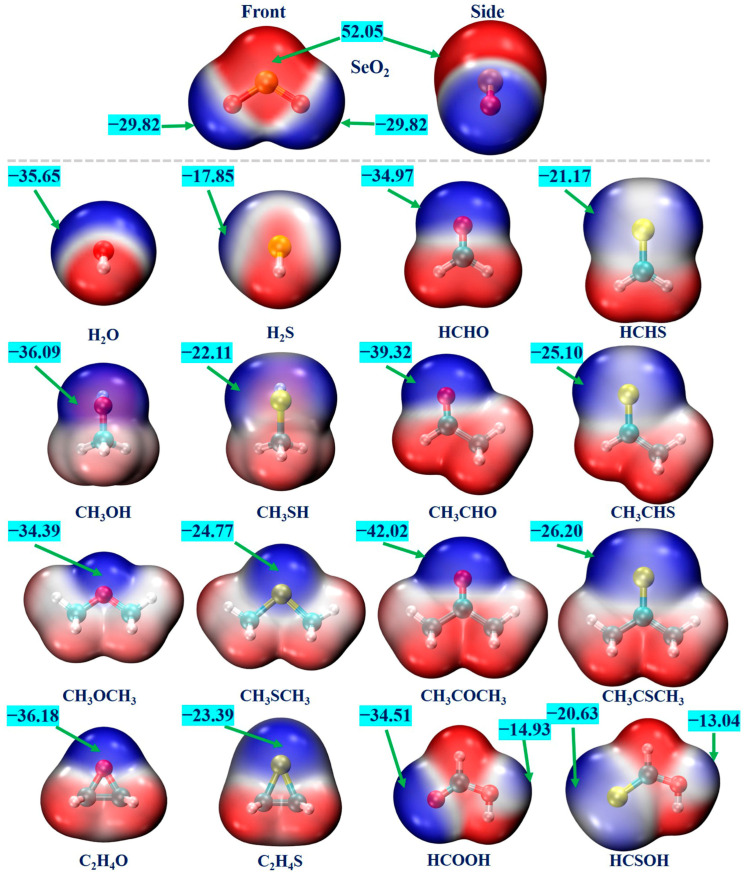
The MEP diagrams of the SeO_2_ and O-/S-containing Lewis bases. The positive and negative electrostatic potentials are represented by the red and blue regions, respectively. The maximum electrostatic potential (*V*_S,max_) and the minimum electrostatic potential (*V*_S,min_) are given in kcal/mol.

MEP is a valuable analytical tool for the prediction and assessment of various non-covalent interactions [63]. We conducted an MEP analysis for each monomer to pinpoint the interaction sites. Figure 1 displays the MEP maps of all monomers, where the red and blue regions denote the positive and negative electrostatic potentials, respectively. One can see that the SeO_2_ molecule has a π–hole region around the Se atom, characterized by the positive-electrostatic-potential areas that are perpendicular to its molecular plane. In addition, there exists one blue region around each O atom, corresponding to the negative electrostatic potentials. The computed most positive and most negative MEP values (*V*_S,max_ and *V*_S,min_) for the SeO_2_ molecule are 52.05 kcal/mol and −29.82 kcal/mol, respectively. The *V*_S,max_ value of the SeO_2_ molecule is significantly larger than that of the SO_2_ molecule (43.74 kcal/mol, calculated at the same level) [45]. This is consistent with the fact that the electronegativity of the S atom is larger than that of the Se atom. The negative-electrostatic-potential regions of water (H_2_O), methanol (CH_3_OH), dimethyl ether (CH_3_OCH_3_), ethylene oxide (C_2_H_4_O), formaldehyde (HCHO), acetaldehyde (CH_3_CHO), acetone (CH_3_COCH_3_), and formic acid (HCOOH) molecules and their corresponding S analogs are related to the lone pairs of the O and S atoms. All of the O- and S-containing Lewis bases possess positive-electrostatic-potential regions surrounding the H atoms. The calculated *V*_S,min_ values of these O-containing molecules range from −34.39 kcal/mol to −42.02 kcal/mol, which are clearly more negative than those of the corresponding S-containing molecules, varying from −17.85 kcal/mol to −26.20 kcal/mol. This difference can be attributed to the smaller electronegativity and bigger polarizability of the S atom compared to those of the O atom. It is interesting to note that the *V*_S,min_ value becomes more negative as the H atoms of the H_2_O, H_2_S, HCHO, and HCHS molecules are gradually replaced by the electron-donating methyl group. For example, the *V*_S,min_ value becomes more negative from −34.97 kcal/mol for the HCHO molecule to −39.32 kcal/mol for the CH_3_CHO molecule and −42.02 kcal/mol for the CH_3_COCH_3_ molecule, and a similar trend was observed for the corresponding S analogs. In addition, it should also be mentioned that the negative MEP values on the O atom of the hydroxyl groups in both HCOOH and HCSOH molecules are −14.93 and −13.04 kcal/mol, respectively. Accordingly, it is predicted that the Se atom within the SeO_2_ molecule can engage in forming Se**∙∙∙**O/S ChBs with the O/S atoms of all Lewis bases, and the O atoms of the SeO_2_ molecule can interact with the H atoms of Lewis bases to form C/O–H**∙∙∙**O hydrogen bonds.

### 2.2. Geometrical Structures and Binding Energies of the Binary Complexes

**Figure 2:**
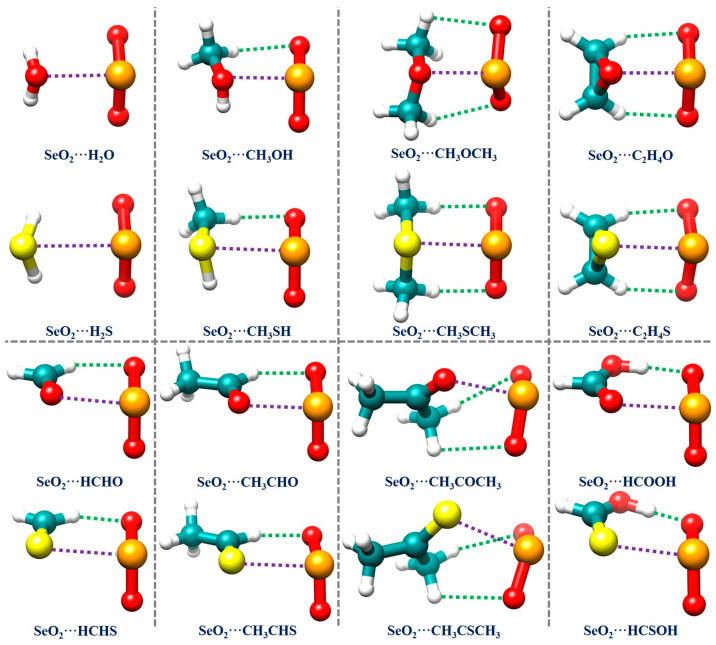
The optimized geometries of the global minima of the studied chalcogen-bonded complexes. The purple and green dotted lines denote the chalcogen bonds and hydrogen bonds, respectively, revealed by conducting an NCIplot analysis.

Because the MEP analysis results reveal that there are relatively few interaction sites in all of the monomers, we directly constructed the initial structures of all of the investigated complexes using chemical intuition. The resulting optimized geometrical structures of the global minima for all 16 studied complexes are displayed in Figure 2. The Cartesian coordinates of these structures are summarized in Table S1 in the Supplementary Materials (SMs). Table 1 also collects the binding energies and several important geometrical parameters related to the Se**∙∙∙**O/S ChBs and C/O–H**∙∙∙**O HBs in all of the investigated complexes. It is observed that the SeO_2_**∙∙∙**H_2_O and SeO_2_**∙∙∙**C_2_H_4_O complexes, along with their S analogs, have *C*_s_ symmetry. Interestingly, the structure of the SeO_2_**∙∙∙**CH_3_OCH_3_ complex possesses *C*_1_ symmetry, whilst its S analog has *C*_s_ symmetry. The remaining complexes exhibit *C*_1_ symmetry.

We also computed the interaction energies (*E*_int_), binding energies (*E*_B_), and deformation energies (*E*_def_) for the studied complexes. All of these data are also summarized in Table 1. It should be pointed out that the *E*_def_ value can be utilized as a measure for assessing the degree of deformation of each component within the complexes. For the Se**∙∙∙**O chalcogen-bonded complexes, the *E*_int_ values range from −4.68 kcal/mol to −10.83 kcal/mol. There exists a fairly wide range of *E*_int_ values between −3.53 kcal/mol and −13.77 kcal/mol for the Se**∙∙∙**S chalcogen-bonded complexes. The *E*_int_ value of each chalcogen-bonded complex is significantly more negative than its *E*_B_ value, varying from −2.42 kcal/mol to −9.64 kcal/mol. This discrepancy is attributed to the distortion of the monomeric structures resulting from the dimerization, with the *E*_def_ value falling within the range of 1.11–6.19 kcal/mol. In the case of the SeO_2_**∙∙∙**HCSOH complex, its *E*_def_ value is the largest, reaching 6.19 kcal/mol. It is found that both the interaction energy and binding energy are greatly affected by the introduction of the methyl and hydroxyl groups. For example, when the H atoms of H_2_O are successively substituted by the methyl groups, the *E*_int_ values exhibit a rapid increase, from −4.68 kcal/mol for the SeO_2_**∙∙∙**H_2_O complex to −7.86 kcal/mol for the SeO_2_**∙∙∙**CH_3_OH complex and −10.12 kcal/mol for the SeO_2_**∙∙∙**CH_3_OCH_3_ complex. Similarly, replacing a H atom of HCHO with a hydroxyl group results in an increase in the *E*_int_ value from −6.81 kcal/mol for the SeO_2_**∙∙∙**HCHO complex to −9.88 kcal/mol for the SeO_2_**∙∙∙**HCOOH complex. The corresponding S analogs also show a similar trend, but with more pronounced changes. This is due to the fact that the methyl and hydroxyl groups serve as electron donors, which can facilitate the transfer of electrons from the O/S atom into the Se atom of SeO_2_. Additionally, it is interesting to note that the *E*_B_ value for the SeO_2_**∙∙∙**CH_3_COCH_3_ complex with the presence of two methyl groups is slightly smaller than that for the SeO_2_**∙∙∙**CH_3_CHO complex, which contains one methyl group. However, the substitution of two H atoms of HCHS with the methyl groups leads to a decrease in *E*_B_ values for the SeO_2_**∙∙∙**CH_3_CSCH_3_ complex compared to those of the SeO_2_**∙∙∙**HCHS complex. This phenomenon is most likely due to the formation of these two complexes causing a noticeable distortion in the acetone and thioacetone structures compared to their isolated gas-phase structures. Furthermore, we found the lack of a strong linear correlation between the Se**∙∙∙**O/S distance and the *E*_int_ or *E*_B_ value (see Figure S1), which can be attributed to the presence of additional C/O–H**∙∙∙**O hydrogen bonds in most of the studied complexes, in addition to the Se**∙∙∙**O/S ChBs.

As can also be seen from Table 1, the Se**∙∙∙**O distances in the Se**∙∙∙**O chalcogen-bonded complexes range from 2.512 Å to 2.765 Å, and the Se**∙∙∙**S distances in the Se**∙∙∙**S chalcogen-bonded complexes vary between 2.818 Å and 3.309 Å. All of the ChB distances (*R*_ChB_) are significantly shorter than the sum (*R*_sum,1_) of the van der Waals radii of two interacting atoms, by 19.2% to 26.5% in the Se**∙∙∙**O chalcogen-bonded complexes and by 10.6% to 23.8% in the Se**∙∙∙**S chalcogen-bonded complexes [64], thus indicating that a strong Se**∙∙∙**O/S ChB is formed in each studied complex. It is noteworthy that the Se**∙∙∙**O distance shortens from 2.765 Å in the SeO_2_**∙∙∙**H_2_O complex to 2.598 Å in the SeO_2_**∙∙∙**CH_3_OH complex and 2.535 Å in the SeO_2_**∙∙∙**CH_3_OCH_3_ complex when the H atoms of H_2_O are gradually replaced by the electron-donating methyl group. A similar trend concerning the Se**∙∙∙**S distance is also observed for the corresponding S analogs. However, this observed trend in the Se**∙∙∙**O/S distance is not evident in the SeO_2_**∙∙∙**HCHO, SeO_2_**∙∙∙**CH_3_CHO, and SeO_2_**∙∙∙**CH_3_COCH_3_ complexes and their S analogs as the methyl group progressively replaces the H atoms of both HCHO and HCHS molecules. Furthermore, the replacement of one H atom with a hydroxyl group in both the HCHO and HCHS molecules results in a shortening of the Se**∙∙∙**O and Se**∙∙∙**S distances in the SeO_2_**∙∙∙**HCOOH and SeO_2_**∙∙∙**HCSOH complexes by 0.030 Å and 0.203 Å, respectively, compared to the SeO_2_**∙∙∙**HCHO and SeO_2_**∙∙∙**HCHS complexes. It is important to note that the π–hole-type Se**∙∙∙**O/S ChBs observed in this work exhibit significantly shorter distances than the σ–hole-type Se**∙∙∙**O/S ChBs found in proteins [13], molecular complexes [65], and protein–ligand complexes [47,55]. Table 1 also illustrates that the O–H**∙∙∙**O HB lengths in the SeO_2_**∙∙∙**HCOOH and SeO_2_**∙∙∙**HCSOH complexes are 1.787 Å and 1.534 Å, respectively, indicating that the strength of the O–H**∙∙∙**O HB in the former is clearly weaker than that in the latter. The C–H**∙∙∙**O HB lengths in the remaining complexes range from 2.181 Å to 2.884 Å. It is noteworthy that the majority of the O**∙∙∙**H HB lengths within all of the investigated complexes are clearly shorter than the sum of the van der Waals radii of the two interacting atoms (2.62 Å) [64]. The computed ∠O–H**∙∙∙**O angle is in the range of 166.2–174.6° and the computed ∠C–H**∙∙∙**O angle varies between 101.0° and 150.6° (see Table 1).

### 2.3. QTAIM Analysis

**Figure 3:**
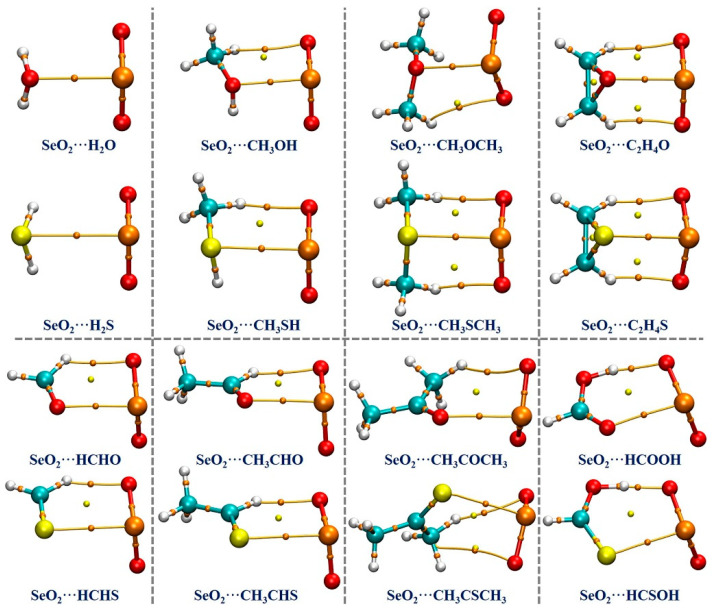
The QTAIM diagrams for the studied complexes. The orange dots indicate (3, −1) critical points, which are called bond critical points (BCPs), and the yellow dots indicate (3, +1) critical points, which are called ring critical points (RCPs). The bond paths (BPs) are indicated by the brown lines.

Based on the optimized geometries at the MP2/aug-cc-pVTZ computational level, we performed a QTAIM analysis to estimate and understand the strength and nature of the intermolecular interactions occurring in the examined complexes. The QTAIM analysis results are graphically shown in Figure 3. It is seen that there exists one (3, −1) critical point, which is referred to as the bond critical point (BCP), and one bond path (BP) between the Se atom of SeO_2_ and the chalcogen atom of the Lewis bases in each of the studied complexes, indicating the formation of π–hole-type Se**∙∙∙**O/S ChBs in all of the complexes under investigation. It should be noted that the BCPs and BPs associated with the O/S–H**∙∙∙**O HBs in both the SeO_2_**∙∙∙**H_2_O and SeO_2_**∙∙∙**H_2_S complexes are not identified, likely due to these H**∙∙∙**O binding distances being significantly longer (about 3.03 Å in the former and 2.98 Å in the latter) compared to the sum of the van der Waals radii of two interacting atoms (2.62 Å). Nevertheless, one or two BCPs and BPs related to the C/O–H**∙∙∙**O HBs are found in all of the remaining complexes.

The topological parameters, including the electron density *ρ*, Laplacian of the electron density ∇^2^*ρ*, the local kinetic energy density *G*, the local potential energy density *V*, and the total energy density *H*, at the BCPs associated with the Se**∙∙∙**O/S ChBs and C/O–H**∙∙∙**O HBs were calculated and are provided in Table 2. It is found that the *ρ* value at the Se**∙∙∙**O BCP varies between 0.0223 a.u for the SeO_2_**∙∙∙**H_2_O complex and 0.0390 a.u for the SeO_2_**∙∙∙**C_2_H_4_O complex, and the *ρ* value at the Se**∙∙∙**S BCP ranges from 0.0151 a.u for the SeO_2_**∙∙∙**H_2_S complex to 0.0410 a.u for the SeO_2_**∙∙∙**HCSOH complex. It should be mentioned that the *ρ* value is linearly correlated with the Se**∙∙∙**O/S binding distance (Figure S2). However, the absence of a good linear relationship between the *ρ* value at the Se**∙∙∙**O/S BCP and the *E*_int_ or *E*_B_ value is observed in Figure S3, which can also be attributed to the existence of additional C/O–H**∙∙∙**O hydrogen bonds. The ∇^2^*ρ* value at the Se**∙∙∙**O BCP in each complex formed by an O-containing Lewis base is significantly larger than that at the Se**∙∙∙**S BCP in the corresponding S-bearing analogs, but all are smaller than 0.1 a.u. The findings suggest that these Se**∙∙∙**O/S ChBs exhibit relatively strong closed-shell interactions due to the presence of positive ∇^2^*ρ* values. Additionally, the Se**∙∙∙**O/S ChBs in the complexes formed by H_2_O, H_2_S, HCHO, CH_3_COCH_3_, and HCOOH are non-covalent in nature, as evidenced by their positive *H* values. Conversely, the Se**∙∙∙**O/S ChBs in all of the other complexes have a partially covalent character, as reflected by their negative *H* values. The *ρ* values at the C/O–H**∙∙∙**O BCP vary from 0.0071 a.u to 0.0670 a.u and the corresponding ∇^2^*ρ* values fall within the range of 0.0275–0.1100 a.u. It is interesting to note that the *H* values are negative in both the SeO_2_**∙∙∙**HCOOH and SeO_2_**∙∙∙**HCSOH complexes, indicating that these O–H**∙∙∙**O HBs are very strong, whilst the *H* values are positive in the remaining complexes, suggesting that these C–H**∙∙∙**O HBs are relatively weak. Furthermore, we also estimated the energy (*E*_NCI_) of each Se**∙∙∙**O/S ChB and C/O–H**∙∙∙**O HB using the following two equations [66,67]:

*E*_NCI_ = 0.375 × *V* − 0.5655 (for the Se**∙∙∙**O/S ChBs)

*E*_NCI_ = −223.08 × *ρ* + 0.7423 (for the C/O–H**∙∙∙**O HBs)

As shown in Table 2, the *E*_NCI_ values of the Se**∙∙∙**O and Se**∙∙∙**S ChBs range from −4.01 kcal/mol to −7.26 kcal/mol and from −2.40 kcal/mol to −6.34 kcal/mol, respectively. It should be pointed out that the *E*_NCI_ value of the Se**∙∙∙**O ChB is significantly larger than that of the Se**∙∙∙**S ChB. The *E*_NCI_ values of the C–H**∙∙∙**O HBs fall within the range of −0.84 kcal/mol to −4.21 kcal/mol. In the SeO_2_**∙∙∙**HCOOH and SeO_2_**∙∙∙**HCSOH complexes, the *E*_NCI_ values of the O–H**∙∙∙**O HB are −7.00 and −14.20 kcal/mol, respectively, which are notably larger than those of the Se**∙∙∙**O/S ChBs within the same complexes. Moreover, the strength of the O–H**∙∙∙**O HB in the SeO_2_**∙∙∙**HCSOH complex is much larger than those of the Se**∙∙∙**O/S ChBs in all of the studied complexes. However, the *E*_NCI_ values of the C–H**∙∙∙**O HBs are smaller than those of the Se**∙∙∙**O/S ChBs in all of the other complexes. It should also be pointed out that the sum of the energies of all identified NCIs varies between −4.01 kcal/mol and −12.15 kcal/mol in each Se**∙∙∙**O chalcogen-bonded complex and between −2.40 kcal/mol and −20.54 kcal/mol in each Se**∙∙∙**S chalcogen-bonded complex. For most of the studied complexes, the sum of these energies is in agreement with the *E*_int_ values. 

Finally, the results in Table 2 also disclose that the addition of the electron-donating methyl and hydroxyl groups had a relatively large impact on the topological properties and strength of the Se**∙∙∙**O/S ChBs. For instance, the *E*_NCI_ value of the Se**∙∙∙**O/S ChB successively increases with the order of H_2_O/H_2_S < CH_3_OH/CH_3_SH < CH_3_OCH_3_/CH_3_SCH_3_. Similar trends can also be observed for the *ρ*, ∇^2^*ρ*, *G*, and *V* values associated with the Se**∙∙∙**O/S ChBs. When one H atom of both HCHO and HCHS was replaced by one hydroxyl group, the *E*_NCI_ value of the Se**∙∙∙**O ChB increased from −4.94 kcal/mol in the SeO2**∙∙∙**HCHO complex to −5.15 kcal/mol in the SeO2**∙∙∙**HCOOH complex, and the *E*_NCI_ value of the Se**∙∙∙**S ChB clearly increased from −4.16 kcal/mol in the SeO2**∙∙∙**HCHS complex to −6.34 kcal/mol in the SeO2**∙∙∙**HCSOH complex.

### 2.4. NCIplot Analysis

**Figure 4:**
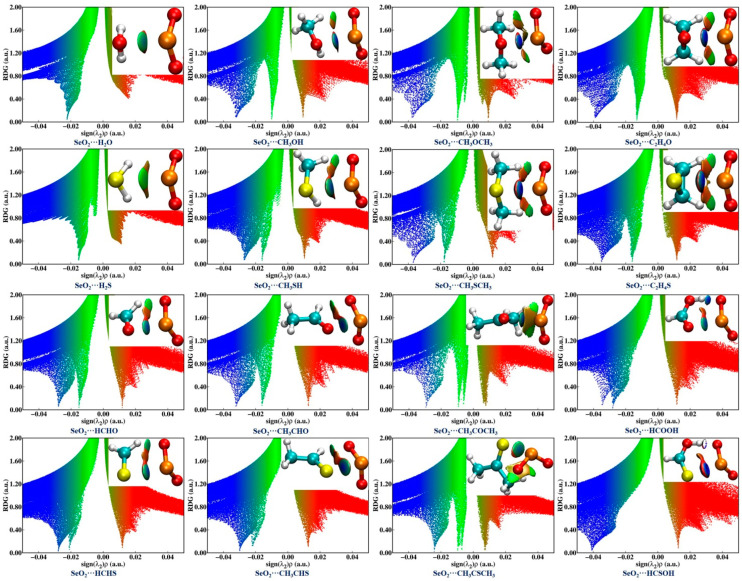
NCI isosurfaces and scatter diagrams of the RDG versus sign(*λ*_2_)*ρ* associated with chalcogen bonds and hydrogen bonds within the sixteen investigated complexes. A 0.55 a.u value was used to create the NCI isosurfaces. The blue and green isosurfaces represent the strongly and weakly attractive interactions, respectively, and red isosurface denotes the repulsive interactions.

The NCIplot method, which is based on the electron density and its reduced density gradient (RGD), was also utilized for characterizing and visualizing the intermolecular interactions in the examined complexes. The NCIplot analysis results are shown in Figure 4, where the blue and green isosurfaces represent the strongly and weakly attractive interactions, respectively, and the red isosurfaces denote the repulsive interactions. It is observed that one green or blue isosurface is present between two chalcogen atoms within the sixteen investigated complexes, providing further evidence of the formation of an attractive ChB. In the case of SeO_2_**∙∙∙**HCSOH, the Se**∙∙∙**S ChB is characterized by one dark-blue isosurface surrounded by one red region, suggesting that this ChB is exceedingly strong. Moreover, the absence of green isosurfaces between the O atoms of SeO_2_ and the H atoms of H_2_O/H_2_S is noted in both the SeO_2_**∙∙∙**H_2_O and SeO_2_**∙∙∙**H_2_S complexes. There is one HB between an O atom of the ChB donor and a H atom of the ChB acceptor, characterized by a green isosurface in the complexes formed by CH_3_OH, CH_3_SH, HCHO, HCHS, CH_3_CHO, and CH_3_CHS, and characterized by a blue isosurface in both the SeO_2_**∙∙∙**HCOOH and SeO_2_**∙∙∙**HCSOH complexes. Two green isosurfaces are observed between two O atoms of SeO_2_ and two H atoms of the Lewis bases in the SeO_2_**∙∙∙**CH_3_OCH_3_, SeO_2_**∙∙∙**C_2_H_4_O, and SeO_2_**∙∙∙**CH_3_COCH_3_ complexes, as well as their S-containing analogs. Interestingly, one C–H**∙∙∙**O BCP unidentified in the QTAIM analysis has a corresponding green isosurface for both the SeO_2_**∙∙∙**CH_3_OCH_3_ and SeO_2_**∙∙∙**CH_3_COCH_3_ complexes in the NCIplot diagrams. These results indicate the existence of weakly/strongly attractive C/O–H**∙∙∙**O HBs within the sixteen studied complexes with the exception of the SeO_2_**∙∙∙**H_2_O and SeO_2_**∙∙∙**H_2_S complexes, which is consistent with the QTAIM analysis outcomes.

The scatter diagrams in Figure 4 depict the relationship between the RDG and the sign of the second eigenvalue *λ*_2_ multiplied by the electron density for each of the sixteen investigated complexes. It should be mentioned that a negative sign(*λ*_2_)*ρ* value represents an attractive interaction, while a positive sign(*λ*_2_)*ρ* value is indicative of a repulsive interaction. Generally, the more negative the sign(*λ*_2_)*ρ* value, the stronger the corresponding interaction strength. One can see that all of the Se**∙∙∙**O/S ChBs and the C/O–H**∙∙∙**O HBs correspond to negative sign(*λ*_2_)*ρ* values, thus corroborating that there are attractive intermolecular interactions in all of these complexes. It is also clearly observed that the sign(*λ*_2_)*ρ* values related to the O–H**∙∙∙**O HBs are more negative than those of the Se**∙∙∙**O/S ChB in both the SeO_2_**∙∙∙**HCOOH and SeO_2_**∙∙∙**HCSOH complexes, suggesting that the strength of the O–H**∙∙∙**O HBs is stronger than that of the Se**∙∙∙**O/S ChBs. Conversely, the C–H**∙∙∙**O HBs are weaker than the Se**∙∙∙**O/S ChBs in all other complexes except for both the SeO_2_**∙∙∙**H_2_O and SeO_2_**∙∙∙**H_2_S complexes as a result of the sign(*λ*_2_)*ρ* values associated with the Se**∙∙∙**O/S ChBs being more negative. It should also be pointed out that the sign(*λ*_2_)*ρ* value of the Se**∙∙∙**S ChB in the SeO_2_**∙∙∙**HCSOH complex is obviously more negative than that of the Se**∙∙∙**O ChB in the SeO_2_**∙∙∙**HCOOH complex, further indicating the Se**∙∙∙**O ChB is weaker than the Se**∙∙∙**S ChB. However, in all of the remaining complexes, the Se**∙∙∙**O ChBs are stronger than the Se**∙∙∙**S ChBs due to the spikes related to the Se**∙∙∙**O ChBs being located at more negative sign(*λ*_2_)*ρ* values. This agrees well with the outcomes of the MEP and QTAIM analyses. Moreover, it is evident that the sign(*λ*_2_)*ρ* value associated with the Se**∙∙∙**O/S ChBs becomes more negative as the number of methyl groups and the number of C–H**∙∙∙**O HBs increase in the complexes of SeO_2_ with R_1_OR_2_ and R_1_CHO (R_1_ = H, CH_3_; R_2_ = H, CH_3_). Substituting a H atom of HCHO and HCHS with a hydroxyl group results in a more negative sign(*λ*_2_)*ρ* value for the Se**∙∙∙**O/S ChBs in both SeO_2_**∙∙∙**HCOOH and SeO_2_**∙∙∙**HCSOH complexes. These results further demonstrate that the introduction of electron-donating methyl and hydroxyl groups and the presence of additional C/O–H**∙∙∙**O HBs have a significant impact on the strength of Se**∙∙∙**O/S ChBs, which is in agreement with the QTAIM analysis results.

### 2.5. NBO Analysis

**Figure 5:**
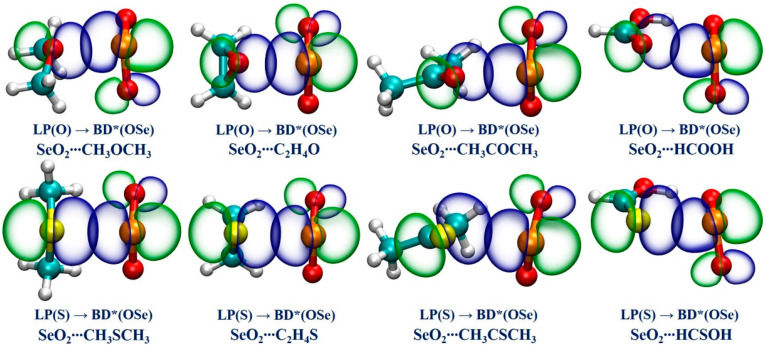
The diagrams of the NBOs associated with the LP(O/S) → BD*(OSe) orbital interactions for the eight selected complexes.

We performed an NBO analysis to gain a deeper understanding of the nature of the Se**∙∙∙**O/S ChBs in all of the studied complexes by examining the donor–acceptor interactions and the associated second-order perturbation energy (*E*^(2)^), which can be used to qualitatively determine a Se**∙∙∙**O/S ChB’s strength. Table 3 collects the NBO analysis findings. It is found that there exists one donor–acceptor interaction between the O/S lone pair of the ChB acceptors and an anti-bonding BD*(OSe) orbital of SeO_2_ in each studied complex. It is evidenced that the *E*^(2)^ values associated with the LP(O)→BD*(OSe) orbital interactions in the Se**∙∙∙**O chalcogen-bonded complexes vary between 5.95 kcal/mol and 15.76 kcal/mol, and the *E*^(2)^ values for the LP(S)→BD*(OSe) orbital interactions range from 7.99 kcal/mol to 56.02 kcal/mol in the Se**∙∙∙**S chalcogen-bonded complexes. Remarkably, in the case of SeO_2_**∙∙∙**HCSOH, the *E*^(2)^ value related to the Se**∙∙∙**S ChB is the largest in magnitude, further demonstrating that this ChB possesses some degree of covalent character. Comparing the results between the complexes of SeO_2_ with the R_1_OR_2_, R_1_SR_2_, R_1_COR_2_, and R_1_CSR_2_ (R_1_ = H, CH_3_, R_2_ = H, CH_3_) indicates that the introduction of the electron-donating methyl group also has a large influence on the *E*^(2)^ value. Furthermore, replacing a H atom of HCHO and HCHS with a hydroxyl group notably increases the *E*^(2)^ value associated with the LP(O/S)→BD*(OSe) orbital interaction in both SeO_2_**∙∙∙**HCOOH and SeO_2_**∙∙∙**HCSOH complexes. These NBO outcomes are highly consistent with the above-mentioned QTAIM and NCIplot findings. Finally, the NBO plots related to the LP(O/S)→BD*(OSe) orbital interactions in the eight selected complexes are displayed in Figure 5. It is observed that the LP(O/S) orbital of the ChB acceptors and the BD*(OSe) anti-bonding orbital of SeO_2_ have a significant overlap, evidencing the formation of strong Se**∙∙∙**O/S ChBs.

### 2.6. SAPT Analysis

The SAPT approach was also employed to decompose the total interaction energies (*E*_total_) of the investigated complexes into electrostatic (*E*_elec_), induction (*E*_ind_), dispersion (*E*_disp_), and exchange–repulsion (*E*_ex-re_) components for a quantitative understanding of the physical nature of intermolecular interactions. Table 4 demonstrates that the electrostatic component is primarily responsible for the stabilization of the complexes under investigation. This component accounts for 51–62% of the total attractive interaction energy in the Se**∙∙∙**O chalcogen-bonded complexes, which is slightly higher than the levels observed in the Se**∙∙∙**S chalcogen-bonded complexes, where it accounts for 47–53% of the total attractive interaction energy. However, the contribution of the induction and dispersion components is also relatively large and unneglectable. The sum of these two components accounts for about 38–59% of the total attractive interaction energy in the Se**∙∙∙**O chalcogen-bonded complexes and about 47–53% of the total attractive interaction energy in the Se**∙∙∙**S chalcogen-bonded complexes. It is interesting to note that the induction component slightly increases its contributions to the total attractive interaction energy in the Se**∙∙∙**S chalcogen-bonded complexes compared with that in the Se**∙∙∙**O chalcogen-bonded complexes. Furthermore, the contribution of the electrostatic component gradually decreases as the number of methyl groups increases in the SeO_2_**∙∙∙**R_1_OR_2_ and SeO_2_**∙∙∙**R_1_SR_2_ complexes (R_1_ = H, CH_3_, R_2_ = H, CH_3_), while the introduction of methyl groups has minimal effects on the electrostatic contribution in the SeO_2_**∙∙∙**R_1_COR_2_ and SeO_2_**∙∙∙**R_1_CSR_2_ complexes (R_1_ = H, CH_3_, R_2_ = H, CH_3_). Compared to the SeO_2_**∙∙∙**HCHO and SeO_2_**∙∙∙**HCHS complexes, the introduction of hydroxyl groups in the SeO_2_**∙∙∙**HCOOH and SeO_2_**∙∙∙**HCSOH complexes has little effect on the electrostatic contribution but does affect the dispersion and induction contributions. One can also see from Table 4 that the total interaction energies vary between −5.32 kcal/mol and −10.06 kcal/mol in the Se**∙∙∙**O chalcogen-bonded complexes and between −3.56 kcal/mol and −12.54 kcal/mol in the Se**∙∙∙**S chalcogen-bonded complexes. Moreover, Figure S4 shows that the *E*_total_ values estimated from the SAPT method exhibit a good correlation with the *E*_int_ values reported in Table 1 for all of the investigated complexes.

## 3. Computational Methods

The geometrical structures of all of the monomers and complexes were optimized by means of the MP2/aug-cc-pVTZ level of theory [68,69]. This approach has been demonstrated to have high accuracy and reliability in the investigation of various chalcogen-bonded complexes [45,49,58,62,70,71]. All of the obtained structures were verified to be true minima without imaginary frequencies by implementing the harmonic vibrational frequency calculations at the same level. The interaction energy (*E*_int_) was computed as the difference in energy between each complex and the sum of energies of the monomers frozen within the complex. This quantity then corresponds to the binding energy (*E*_B_) by utilizing the optimized monomeric geometries. Both the *E*_int_ and *E*_B_ values were also corrected by considering the basis set superposition error (BSSE), employing the counterpoise method [72]. All computations were executed using the Gaussian 16 software [73].

The MEP of each of the monomers on the 0.001 electron/Bohr^3^ isosurface were computed at the MP2/aug-cc-pVTZ level utilizing the Multiwfn program [74], and the VMD software (version 1.9.3) [75] was employed to visualize the MEP maps. The QTAIM approach [76] at the MP2/aug-cc-pVTZ level implemented in the Multiwfn program was utilized to identify the bond paths (BPs) and bond critical points (BCPs) within the studied complexes and estimate the topological properties at each BCP. For the sake of characterizing the Se**∙∙∙**O/S ChB within each studied complex, the NCIplot analysis [77] was conducted with the Multiwfn program and the results of the corresponding NCIplot analysis were visualized by means of both VMD and Gnuplot [78] programs. The *E*^(2)^ values associated with the orbital interactions between the SeO_2_ and various Lewis bases in the studied complexes was evaluated at the B3LYP-D3(BJ)/aug-cc-pVTZ level by conducting an NBO analysis [79] with the NBO 3.1 module incorporated into the Gaussian16 program. The SAPT analysis [80] was performed at the SAPT2 + (CCD)δMP2/aug-cc-pVTZ level to estimate the contributions of different attractive and repulsive components to the total binding energies of all of the studied complexes by employing the PSI4 software (version 1.3.2) [81].

## 4. Conclusions

In this work, the selenium-centered chalcogen bonds between SeO_2_ and a series of O/S-containing Lewis bases were computationally investigated at the MP2/aug-cc-pVTZ level of theory. Various computational methods, including the MEP, QTAIM, NCIplot, NBO, and SAPT methods, were also utilized to characterize and understand the strength and nature of such ChBs. The MEP analysis results reveal that the positive-electrostatic-potential regions around the Se atom of SeO_2_ exhibit attractive interactions with the negative-electrostatic-potential regions around the O/S atom of various Lewis bases, resulting in the formation of Se**∙∙∙**O/S ChBs. The Se**∙∙∙**O binding distances vary between 2.512 Å and 2.765 Å and the Se**∙∙∙**S binding distances range from 2.818 Å to 3.309 Å, both of which are significantly shorter than the sum of the van der Waals radii of the two interacting atoms. The interaction energy varies between −4.68 kcal/mol and −10.83 kcal/mol for the Se**∙∙∙**O chalcogen-bonded complexes and ranges from −3.53 kcal/mol to −13.77 kcal/mol for the Se**∙∙∙**S chalcogen-bonded complexes. The closed-shell nature of all of these Se**∙∙∙**O/S ChBs was corroborated by conducting a QTAIM analysis, showing that the Se**∙∙∙**O/S ChBs have a partially covalent character in most chalcogen-bonded complexes. Both the QTAIM and NCIplot analyses demonstrate that the Se**∙∙∙**O ChB in the SeO_2_**∙∙∙**HCOOH complex is weaker than the Se**∙∙∙**S ChB in the SeO_2_**∙∙∙**HCSOH complex, whilst the former is stronger than the latter in all of the remaining complexes. Furthermore, our findings also disclose that the O–H**∙∙∙**O HBs are stronger than the Se**∙∙∙**O/S ChBs in both SeO_2_**∙∙∙**HCOOH and SeO_2_**∙∙∙**HCSOH complexes, while the C–H**∙∙∙**O HBs are weaker than the Se**∙∙∙**O/S ChBs in all other complexes. The NBO analysis demonstrates that the interaction between the LP at the O/S atoms of Lewis bases and the BD*(OSe) anti-bonding orbital of SeO_2_ is the primary stabilizing factor for all of the investigated complexes except for SeO_2_**∙∙∙**HCSOH. Conversely, the greatest contributor to the stabilization of the latter complex is the interaction between the LP(O) orbital of SeO_2_ and the BD*(OH) anti-bonding orbital of HCSOH. The SAPT calculations also uncover that the electrostatic component makes the most significant contribution to the stabilization of the examined complexes, while the induction and dispersion contributions are also relatively large and unneglectable. It is worth highlighting that the introduction of electron-donating methyl and hydroxyl groups has a significant impact on the strength of both Se**∙∙∙**O and Se**∙∙∙**S ChBs. Furthermore, the existence of additional C/O–H**∙∙∙**O interactions can also affect the geometric structures and strength of Se**∙∙∙**O**/**S ChBs. We hope that the results of this study will provide significant value in the fields of organocatalysis, crystal engineering, molecular recognition, and materials science.

## Figures and Tables

**Table 1:** The interaction energies (*E*_int_), binding energies (*E*_B_), and deformation energies (*E*_def_) together with geometrical data related to the Se**∙∙∙**O/S ChBs and C/O–H**∙∙∙**O HBs within the investigated complexes.

Complex	Symmetry	*E*_int_ ^a^ (kcal/mol)	*E*_B_ ^b^ (kcal/mol)	*E*_def_ (kcal/mol)	NCIs	*R*_NCI_ ^c^ (Å)	*R*_sum,1_ ^d^ (Å)
SeO_2_**∙∙∙**H_2_O	*C*_s_	−4.68	−3.39	1.28	Se**∙∙∙**O	2.765	3.42 (19.2%) ^e^
SeO_2_**∙∙∙**CH_3_OH	*C*_1_	−7.86	−5.37	2.49	Se**∙∙∙**O	2.598	3.42 (24.0%)
					C–H**∙∙∙**O	2.490 (116.9°) ^f^	2.62 (5.0%)
SeO_2_**∙∙∙**CH_3_OCH_3_	*C*_1_	−10.12	−6.73	3.39	Se**∙∙∙**O	2.535	3.42 (25.9%)
					C–H**∙∙∙**O_U_^g^	2.598 (106.0°)	2.62 (0.8%)
					C–H**∙∙∙**O_L_^g^	2.550 (114.3°)	2.62 (2.7%)
SeO_2_**∙∙∙**C_2_H_4_O	*C*_s_	−10.83	−7.72	3.11	Se**∙∙∙**O	2.512	3.42 (26.5%)
					C–H**∙∙∙**O ^h^	2.340 (124.7°)	2.62 (10.7%)
SeO_2_**∙∙∙**HCHO	*C*_1_	−6.81	−4.70	2.11	Se**∙∙∙**O	2.672	3.42 (21.9%)
					C–H**∙∙∙**O	2.261 (123.0°)	2.62 (13.7%)
SeO_2_**∙∙∙**CH_3_CHO	*C*_1_	−8.50	−6.00	2.50	Se**∙∙∙**O	2.602	3.42 (23.9%)
					C–H**∙∙∙**O	2.237 (124.3°)	2.62 (14.6%)
SeO_2_**∙∙∙**CH_3_COCH_3_	*C*_1_	−9.08	−5.83	3.25	Se**∙∙∙**O	2.631	3.42 (23.1%)
					C–H**∙∙∙**O_U_	2.512 (144.4°)	2.62 (4.1%)
					C–H**∙∙∙**O_L_	2.884 (101.0°)	2.62 (10.1%)
SeO_2_**∙∙∙**HCOOH	*C*_1_	−9.88	−6.57	3.31	Se**∙∙∙**O	2.642	3.42 (22.7%)
					O–H**∙∙∙**O	1.787 (166.2°)	2.62 (31.8%)
SeO_2_**∙∙∙**H_2_S	*C*_s_	−3.53	−2.42	1.11	Se**∙∙∙**S	3.309	3.70 (10.6%)
SeO_2_**∙∙∙**CH_3_SH	*C*_1_	−7.80	−5.47	2.33	Se**∙∙∙**S	3.029	3.70 (18.1%)
					C–H**∙∙∙**O	2.206 (139.2°)	2.62 (15.8%)
SeO_2_**∙∙∙**CH_3_SCH_3_	*C*_s_	−13.42	−9.64	3.78	Se**∙∙∙**S	2.884	3.70 (22.1%)
					C–H**∙∙∙**O	2.182 (131.1°)	2.62 (16.7%)
SeO_2_**∙∙∙**C_2_H_4_S	*C*_s_	−12.21	−8.90	3.31	Se**∙∙∙**S	2.906	3.70 (21.5%)
					C–H∙∙∙O	2.211 (136.9°)	2.62 (15.6%)
SeO_2_**∙∙∙**HCHS	*C*_1_	−7.67	−5.55	2.12	Se**∙∙∙**S	3.021	3.70 (18.4%)
					C–H**∙∙∙**O	2.076 (137.1°)	2.62 (20.8%)
SeO_2_**∙∙∙**CH_3_CHS	*C*_1_	−9.14	−6.71	2.43	Se**∙∙∙**S	2.982	3.70 (19.4%)
					C–H**∙∙∙**O	2.059 (140.1°)	2.62 (21.4%)
SeO_2_**∙∙∙**CH_3_CSCH_3_	*C*_1_	−7.92	−5.01	2.91	Se**∙∙∙**S	3.090	3.70 (16.5%)
					C–H**∙∙∙**O_U_	2.502 (150.6°)	2.62 (4.5%)
					C–H**∙∙∙**O_L_	2.738 (106.2°)	2.62 (4.5%)
SeO_2_**∙∙∙**HCSOH	*C*_1_	−13.77	−7.57	6.20	Se**∙∙∙**S	2.818	3.70 (23.8%)
					O–H**∙∙∙**O	1.534 (174.6°)	2.62 (41.5%)

^a^ *E*_int_ indicates the interaction energy including the zero-point energy (ZPE) and BSSE corrections. ^b^ *E*_B_ indicates the binding energy including the zero-point energy (ZPE) and BSSE corrections. ^c^ *R*_NCI_ denotes the distances of the chalcogen bonds and hydrogen bonds. ^d^ *R*_sum,1_ represents the sum of the van der Waals radii of the two interacting atoms. ^e^ The parenthesized values indicate the percent difference computed by using the following equation: (|*R*_NCI_ − *R*_sum,1_|/*R*_sum,1_) × 100%. ^f^ The parenthesized values represent the ∠C/O–H**∙∙∙**O angle. ^g^ O_U_ and O_L_ represent the upper and lower O atoms of SeO_2_ in Figure 2, respectively. ^h^ Both the lengths and angles of the two C–H**∙∙∙**O hydrogen bonds in these complexes are identical due to them having *C*_s_ symmetry.

**Table 2:** The energies (*E*_NCI_) of chalcogen bonds and hydrogen bonds, as well as the electron density *ρ* and its Laplacian ∇^2^*ρ*, the local kinetic energy density *G*, the local potential energy density *V*, and the total energy density *H* at the Se**∙∙∙**O/S and C/O–H**∙∙∙**O (3, −1) critical points, which are called bond critical points (BCPs), in each studied complex.

Complex	BCPs	*E*_NCI_ (kcal/mol)	*ρ* (a.u)	∇^2^*ρ* (a.u)	*G* (a.u)	*V* (a.u)	*H* (a.u)
SeO_2_**∙∙∙**H_2_O	Se**∙∙∙**O	−4.01 ^b^	0.0223	0.0666	0.0156	−0.0146	0.0010
SeO_2_**∙∙∙**CH_3_OH	Se**∙∙∙**O	−5.95 ^b^	0.0324	0.0852	0.0221	−0.0229	−0.0008
	C–H**∙∙∙**O	−1.51 ^c^	0.0101	0.0384	0.0080	−0.0063	0.0016
SeO_2_**∙∙∙**CH_3_OCH_3_	Se**∙∙∙**O	−6.99 ^b^	0.0377	0.0911	0.0250	−0.0273	−0.0023
	C–H**∙∙∙**O	−1.38 ^c^	0.0095	0.0365	0.0077	−0.0062	0.0015
SeO_2_**∙∙∙**C_2_H_4_O	Se**∙∙∙**O	−7.26 ^b^	0.0390	0.0945	0.0260	−0.0285	−0.0024
	C–H**∙∙∙**O_U_^a^	−2.20 ^c^	0.0132	0.0501	0.0105	−0.0084	0.0021
	C–H**∙∙∙**O_L_^a^	−2.20 ^c^	0.0132	0.0501	0.0105	−0.0084	0.0021
SeO_2_**∙∙∙**HCHO	Se**∙∙∙**O	−4.94 ^b^	0.0280	0.0761	0.0188	−0.0186	0.0002
	C–H**∙∙∙**O	−2.65 ^c^	0.0152	0.0583	0.0123	−0.0099	0.0023
SeO_2_**∙∙∙**CH_3_CHO	Se**∙∙∙**O	−5.79 ^b^	0.0323	0.0839	0.0216	−0.0222	−0.0006
	C–H**∙∙∙**O	−2.87 ^c^	0.0162	0.0613	0.0130	−0.0107	0.0023
SeO_2_**∙∙∙**CH_3_COCH_3_	Se**∙∙∙**O	−5.34 ^b^	0.0294	0.0822	0.0204	−0.0203	0.0001
	C–H**∙∙∙**O	−1.33 ^c^	0.0093	0.0321	0.0068	−0.0055	0.0012
SeO_2_**∙∙∙**HCOOH	Se**∙∙∙**O	−5.15 ^b^	0.0281	0.0807	0.0198	−0.0195	0.0003
	O–H**∙∙∙**O	−7.00 ^c^	0.0347	0.0975	0.0292	−0.0339	−0.0048
SeO_2_**∙∙∙**H_2_S	Se**∙∙∙**S	−2.40 ^b^	0.0151	0.0322	0.0079	−0.0078	0.0001
SeO_2_**∙∙∙**CH_3_SH	Se**∙∙∙**S	−4.10 ^b^	0.0271	0.0415	0.0127	−0.0150	−0.0023
	C–H**∙∙∙**O	−2.85 ^c^	0.0161	0.0603	0.0128	−0.0105	0.0023
SeO_2_**∙∙∙**CH_3_SCH_3_	Se**∙∙∙**S	−5.55 ^b^	0.0371	0.0420	0.0159	−0.0212	−0.0053
	C–H**∙∙∙**O_U_^a^	−3.25 ^c^	0.0179	0.0668	0.0143	−0.0119	0.0024
	C–H**∙∙∙**O_L_^a^	−3.25 ^c^	0.0179	0.0668	0.0143	−0.0119	0.0024
SeO_2_**∙∙∙**C_2_H_4_S	Se**∙∙∙**S	−5.30 ^b^	0.0351	0.0435	0.0155	−0.0201	−0.0046
	C–H**∙∙∙**O_U_^a^	−2.89 ^c^	0.0163	0.0608	0.0130	−0.0107	0.0022
	C–H**∙∙∙**O_L_^a^	−2.89 ^c^	0.0163	0.0608	0.0130	−0.0107	0.0022
SeO_2_**∙∙∙**HCHS	Se**∙∙∙**S	−4.16 ^b^	0.0279	0.0418	0.0129	−0.0153	−0.0024
	C–H**∙∙∙**O	−3.99 ^c^	0.0212	0.0788	0.0173	−0.0150	0.0023
SeO_2_**∙∙∙**CH_3_CHS	Se**∙∙∙**S	−4.49 ^b^	0.0300	0.0427	0.0137	−0.0167	−0.0030
	C–H**∙∙∙**O	−4.21 ^c^	0.0222	0.0808	0.0181	−0.0160	0.0021
SeO_2_**∙∙∙**CH_3_CSCH_3_	Se**∙∙∙**S	−3.63 ^b^	0.0242	0.0400	0.0115	−0.0130	−0.0015
	C–H**∙∙∙**O	−1.42 ^c^	0.0097	0.0328	0.0070	−0.0058	0.0012
	C–H**∙∙∙**O	−0.84 ^c^	0.0071	0.0275	0.0056	−0.0043	0.0013
SeO_2_**∙∙∙**HCSOH	Se**∙∙∙**S	−6.34 ^b^	0.0410	0.0440	0.0178	−0.0245	−0.0068
	O–H**∙∙∙**O	−14.20 ^c^	0.0670	0.1100	0.0532	−0.0790	−0.0257

^a^ Two C–H**∙∙∙**O hydrogen bonds in these complexes are identical and share the same energy and topological parameters due to them having *C*s symmetry. O_U_ and O_L_ represent the upper and lower O atoms of SeO_2_ in Figure 3, respectively. ^b^ Energetic values obtained employing the equation *E*_NCI_ = 0.375 × *V* − 0.5655 from reference [66]. ^c^ Energetic values obtained employing the equation *E*_NCI_ = −223.08 × *ρ* + 0.7423 from reference [67].

**Table 3:** The estimated *E*^(2)^ values (kcal/mol) associated with the Se**∙∙∙**O/S ChBs for all of the investigated complexes according to the NBO scheme at the B3LYP-D3(BJ)/aug-cc-pVTZ level ^a^.

Complex	Donor	Acceptor	*E* ^(2)^	Complex	Donor	Acceptor	*E* ^(2)^
SeO_2_**∙∙∙**H_2_O	LP(O)	BD*(OSe)	5.95	SeO_2_**∙∙∙**H_2_S	LP(S)	BD*(OSe)	7.99
SeO_2_**∙∙∙**CH_3_OH	LP(O)	BD*(OSe)	11.02	SeO_2_**∙∙∙**CH_3_SH	LP(S)	BD*(OSe)	17.83
SeO_2_**∙∙∙**CH_3_OCH_3_	LP(O)	BD*(OSe)	14.87	SeO_2_**∙∙∙**CH_3_SCH_3_	LP(S)	BD*(OSe)	29.00
SeO_2_**∙∙∙**C_2_H_4_O	LP(O)	BD*(OSe)	15.76	SeO_2_**∙∙∙**C_2_H_4_S	LP(S)	BD*(OSe)	26.00
SeO_2_**∙∙∙**HCHO	LP(O)	BD*(OSe)	9.35	SeO_2_**∙∙∙**HCHS	LP(S)	BD*(OSe)	16.38
SeO_2_**∙∙∙**CH_3_CHO	LP(O)	BD*(OSe)	12.46	SeO_2_**∙∙∙**CH_3_CHS	LP(S)	BD*(OSe)	19.27
SeO_2_**∙∙∙**CH_3_COCH_3_	LP(O)	BD*(OSe)	13.09	SeO_2_**∙∙∙**CH_3_CSCH_3_	LP(S)	BD*(OSe)	16.01
SeO_2_**∙∙∙**HCOOH	LP(O)	BD*(OSe)	15.53	SeO_2_**∙∙∙**HCSOH	LP(S)	BD*(OSe)	56.02

^a^ LP and BD* indicate lone pair and anti-bonding orbital, respectively.

**Table 4:** The decomposition results (kcal/mol) of the total interaction energies for the investigated complexes calculated at the SAPT2 + (CCD)δMP2/aug-cc-pVTZ level of theory.

Complex	*E*_elec_	*E*_ind_	*E*_disp_	*E*_ex-re_	*E*_total_
SeO_2_**∙∙∙**H_2_O	−12.86 (62%) ^a^	−3.34 (16%)	−4.66 (22%)	15.53	−5.32
SeO_2_**∙∙∙**CH_3_OH	−20.39 (56%)	−7.52 (21%)	−8.45 (23%)	28.83	−7.54
SeO_2_**∙∙∙**CH_3_OCH_3_	−23.56 (53%)	−9.88 (22%)	−11.41 (25%)	35.29	−9.56
SeO_2_**∙∙∙**C_2_H_4_O	−25.02 (52%)	−11.75 (24%)	−11.65 (24%)	38.55	−9.86
SeO_2_**∙∙∙**HCHO	−15.56 (52%)	−6.90 (23%)	−7.55 (25%)	23.15	−6.85
SeO_2_**∙∙∙**CH_3_CHO	−19.12 (52%)	−8.96 (24%)	−8.71 (24%)	28.76	−8.02
SeO_2_**∙∙∙**CH_3_COCH_3_	−18.83 (54%)	−6.97 (20%)	−9.01 (26%)	26.52	−8.29
SeO_2_**∙∙∙**HCOOH	−22.80 (51%)	−12.06 (27%)	−9.93 (22%)	35.51	−9.28
SeO_2_**∙∙∙**H_2_S	−8.41 (53%)	−2.56 (16%)	−4.88 (31%)	12.28	−3.56
SeO_2_**∙∙∙**CH_3_SH	−18.40 (49%)	−9.27 (25%)	−9.76 (26%)	30.54	−6.90
SeO_2_**∙∙∙**CH_3_SCH_3_	−28.43 (47%)	−17.59 (29%)	−14.63 (24%)	48.28	−12.38
SeO_2_**∙∙∙**C_2_H_4_S	−26.48 (47%)	−15.55 (28%)	−13.78 (25%)	44.24	−11.56
SeO_2_**∙∙∙**HCHS	−18.45 (47%)	−11.03 (28%)	−9.92 (25%)	32.38	−7.02
SeO_2_**∙∙∙**CH_3_CHS	−20.99 (47%)	−12.71 (29%)	−10.79 (24%)	36.28	−8.21
SeO_2_**∙∙∙**CH_3_CSCH_3_	−16.43 (48%)	−7.75 (23%)	−9.79 (29%)	26.28	−7.70
SeO_2_**∙∙∙**HCSOH	−43.82 (47%)	−32.00 (35%)	−16.96 (18%)	80.24	−12.54

^a^ The values enclosed within parentheses represent the contribution of individual attractive component to the total attractive interaction energy.

## Data Availability

The data that support the findings of this study are available within the article and its Supplementary Materials.

## Supplementary Materials

The following supporting information can be downloaded at: https://www.mdpi.com/article/10.3390/ijms25115609/s1.
