# Supplementary material for: Unveiling the Nature and Strength of Selenium-Centered Chalcogen Bonds in Binary Complexes of SeO2 with Oxygen-/Sulfur-Containing Lewis Bases: Insights from Theoretical Calculations
Source: Int J Mol Sci. 2024 May 21;25(11):5609. doi: 10.3390/ijms25115609 (PMC11171880; doi:10.3390/ijms25115609)
Supplement: Supplementary file 1 [file ijms-25-05609-s001.zip › ijms-2972924-supplementary.pdf]

Unveiling the Nature and Strength of Selenium-Centered Chalcogen Bonds in Binary Complexes of SeO_2 with Oxygen-/Sulfur-Containing Lewis Bases: Insights from Theoretical Calculations

Tao Lu, Renhua Chen, Qingyu Liu, Yeshuang Zhong, Fengying Lei * and Zhu Zeng *

School of Basic Medical Sciences/School of Biology and Engineering, Guizhou Medical University, Guiyang 550025, China; lutao0409@gmc.edu.cn (T.L.); chenrenhua5288@163.com (R.C.); l.qingyu@foxmail.com (Q.L.); zhongyeshuang@foxmail.com (Y.Z.)
* Correspondence: leifengying@gmc.edu.cn (F.L.); zengzhu@gmc.edu.cn (Z.Z.)

Contents:

Supplementary figures

Figure S1. Correlation plots between the Se···O/S distance in each studied complex and the corresponding interaction energy (left) and binding energy (right).
Figure S2. Correlation plots between the Se···O/S distance and the electron density ρ at the Se···O/S BCP in each studied complex.
Figure S3. Correlation plots between the electron density ρ at the Se···O/S BCP in each studied complex and the corresponding interaction energy (left) and binding energy (right).
Figure S4. Correlation plots between the interaction energies and the total interaction energies of the studied complexes obtained by the SAPT analysis.

Supplementary tables

Table S1. Cartesian coordinates of the complexes of SeO_2 with O-containing Lewis bases in its principal inertial axis system at the MP2/aug-cc-pVTZ level of theory.

Supplementary figures

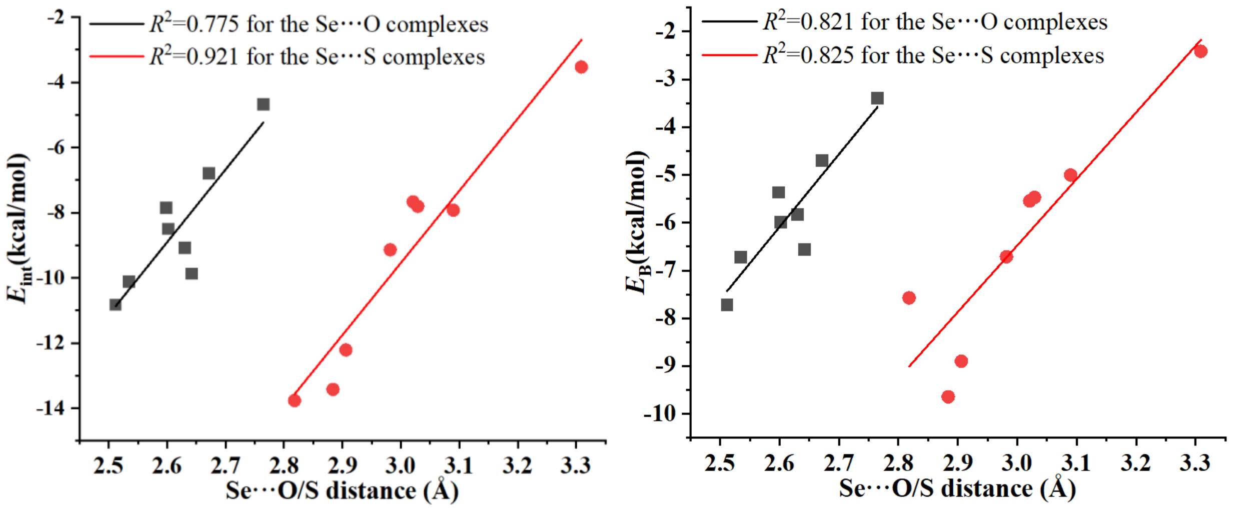

Figure S1. Correlation plots between the Se···O/S distance in each studied complex and the corresponding interaction energy (left) and binding energy (right).

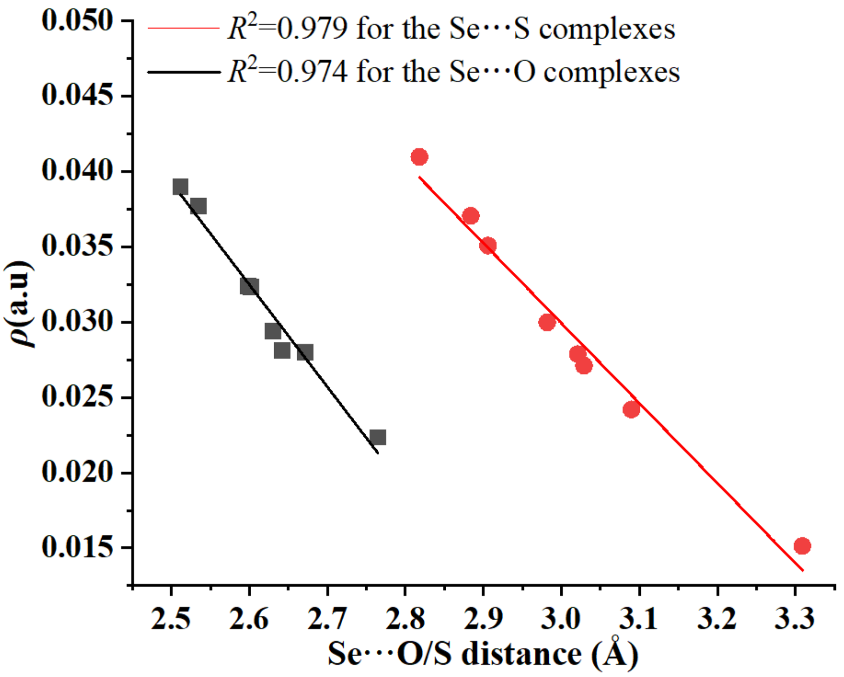

Figure S2. Correlation plots between the Se···O/S distance and the electron density ρ at the Se···O/S BCP in each studied complex.

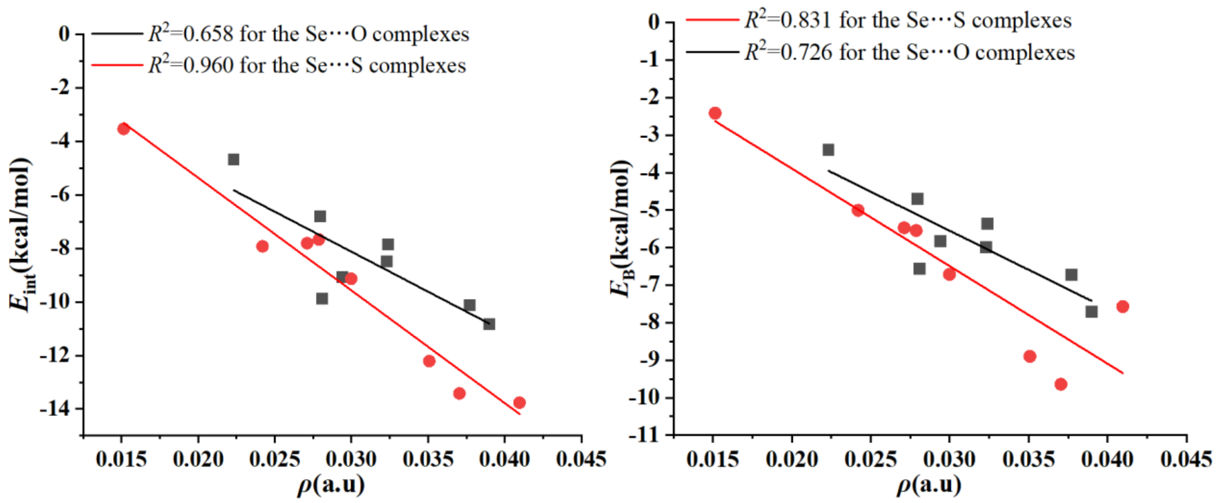

Figure S3. Correlation plots between the electron density ρ at the Se···O/S BCP in each studied complex and the corresponding interaction energy (left) and binding energy (right).

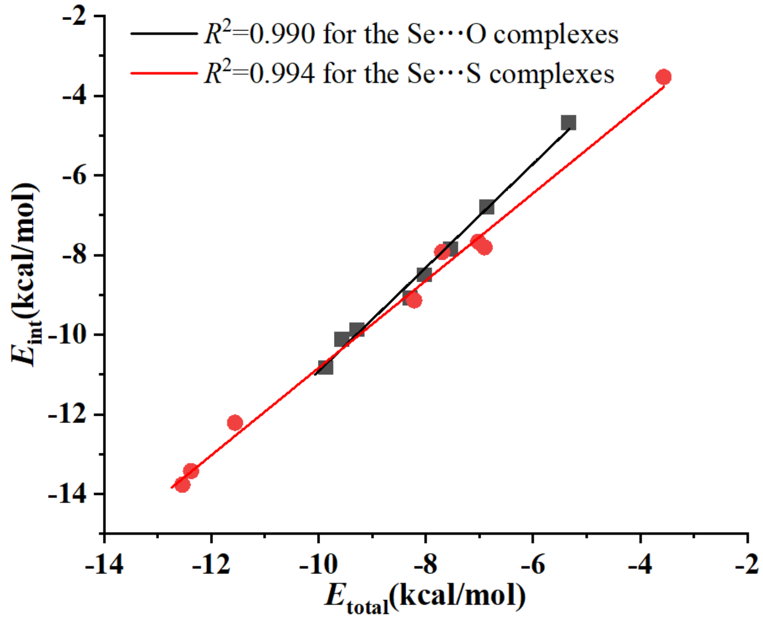

Figure S4. Correlation plots between the interaction energies and the total interaction energies of the studied complexes obtained by the SAPT analysis.

Supplementary tables

Table S1. Cartesian coordinates of the complexes of SeO_2 with O-containing Lewis bases in its principal inertial axis system at the MP2/aug-cc-pVTZ level of theory.

**$SeO_2\cdots H_2O$**				**$SeO_2\cdots H_2S$**			
Atom	a (Å)	b (Å)	c (Å)	Atom	*a* (Å)	*b* (Å)	*c* (Å)
O	-2.311492	-0.000053	-0.096557	S	2.450820	-0.005008	-0.075321
H	-2.455003	0.762414	0.476028	H	2.041999	0.964017	0.752728
H	-2.455070	-0.762157	0.476494	H	2.052767	-0.963737	0.769705
O	0.513139	-1.356098	0.597193	O	-0.884389	1.359010	0.597050
O	0.513094	1.356101	0.597214	O	-0.909743	-1.355872	0.596036
Se	0.446828	0.000005	-0.286333	Se	-0.851613	0.001610	-0.290058
$SeO_2\cdots CH_3OH$				$SeO_2\cdots CH_3SH$			
Atom	*a* (Å)	*b* (Å)	*c* (Å)	Atom	*a* (Å)	*b* (Å)	*c* (Å)
C	2.516518	0.279507	0.206540	C	2.273724	0.913489	0.440874
H	2.789513	0.956447	-0.596371	H	2.778351	1.490320	-0.329426
H	3.418328	-0.158890	0.630023	H	2.936470	0.788585	1.291166
H	1.970567	0.832348	0.971232	H	1.351335	1.417159	0.728793
O	1.690587	-0.736121	-0.383257	S	1.905189	-0.698769	-0.300119
H	1.527817	-1.416870	0.283264	H	1.482489	-1.304022	0.817657
O	-0.387007	1.443193	0.454510	O	-1.359900	-0.953857	1.025735
O	-1.074335	-1.156392	0.770002	O	-0.761279	1.518780	0.101046
Se	-0.783509	0.050132	-0.272277	Se	-1.050134	-0.035649	-0.275464
$SeO_2\cdots CH_3OCH_3$				$SeO_2\cdots CH_3SCH_3$			
Atom	*a* (Å)	*b* (Å)	*c* (Å)	Atom	*a* (Å)	*b* (Å)	*c* (Å)
O	1.528328	0.229060	-0.525227	C	1.818517	1.376809	0.489280
C	1.777153	1.217891	0.474410	H	1.891316	2.284812	-0.104334
H	1.217730	2.105060	0.194399	H	0.932520	1.433669	1.125125
H	1.438070	0.864598	1.451730	H	2.723128	1.249269	1.079666
H	2.844977	1.438839	0.511336	C	1.821139	-1.376229	0.487292
C	2.179089	-0.996736	-0.196819	H	0.933376	-1.437628	1.120301
H	1.796320	-1.390782	0.747650	H	1.899593	-2.282896	-0.107640
H	1.967533	-1.697295	-1.000009	H	2.723424	-1.245869	1.080601
H	3.256070	-0.835991	-0.124386	S	1.666317	0.000972	-0.661403
O	-1.273819	1.375702	0.083362	O	-1.191396	1.349420	0.628052
O	-0.712459	-1.076418	1.077037	O	-1.189207	-1.349063	0.630691
Se	-0.958663	-0.177591	-0.250813	Se	-1.192869	-0.000684	-0.280607
$SeO_2\cdots C_2H_4O$				$SeO_2\cdots C_2H_4S$			
Atom	*a* (Å)	*b* (Å)	*c* (Å)	Atom	*a* (Å)	*b* (Å)	*c* (Å)
C	2.145473	0.732751	0.282120	C	-1.921360	-0.699902	0.793916

C	2.152851	-0.729749	0.266608
O	1.485497	0.009386	-0.789537
H	1.457877	1.243176	0.946016
H	3.017210	1.280047	-0.046598
H	1.470017	-1.260368	0.919572
H	3.030035	-1.261116	-0.073583
O	-0.843920	-1.348132	0.637230
O	-0.863544	1.349485	0.628123
Se	-0.970275	-0.003108	-0.260127

SeO_2···HCHO

Atom	*a* (Å)	*b* (Å)	*c* (Å)
C	2.448815	0.115638	0.315156
O	1.868502	-0.648876	-0.435611
H	3.519892	-0.005211	0.514132
H	1.912981	0.940125	0.806579
O	-1.107112	-1.084461	0.838476
O	-0.248598	1.437510	0.368913
Se	-0.712591	0.021702	-0.276055

SeO_2···CH_3CHO

Atom	*a* (Å)	*b* (Å)	*c* (Å)
C	1.991639	0.123309	0.058447
H	1.481962	0.940513	0.596519
C	3.464820	-0.003862	0.236189
H	3.859659	-0.830318	-0.346306
H	3.939906	0.933564	-0.056363
H	3.678334	-0.148614	1.296425
O	1.330243	-0.626479	-0.645451
O	-0.684040	1.447315	0.360143
O	-1.433487	-1.090369	0.907265
Se	-1.158834	0.016013	-0.242169

SeO_2···CH_3COCH_3

Atom	*a* (Å)	*b* (Å)	*c* (Å)
C	2.006979	-0.068741	-0.101744
C	3.451708	-0.453469	0.032407
H	3.562474	-1.533097	0.016621
H	4.013505	-0.009571	-0.791082
H	3.858487	-0.040182	0.956027
O	1.152019	-0.919539	-0.329492
C	1.663094	1.382024	0.078223
H	1.480192	1.551829	1.141920
H	2.489140	2.019857	-0.229016
H	0.756792	1.637814	-0.463422
O	-1.119275	0.341802	1.452306
O	-1.499551	0.799576	-1.181589

C	-1.945527	0.775750	0.702040
H	-1.034320	-1.166039	1.211830
H	-2.855238	-1.230857	0.910477
H	-1.073861	1.316168	1.058485
H	-2.896457	1.286362	0.753811
S	-1.659168	-0.059430	-0.892673
O	1.076481	1.368560	0.603950
O	1.121435	-1.320675	0.686048
Se	1.177193	-0.002733	-0.263163

SeO_2···HCHS

Atom	*a* (Å)	*b* (Å)	*c* (Å)
C	-2.180571	0.811795	0.471782
H	-3.145773	1.127814	0.854125
H	-1.332961	1.494002	0.572568
S	-1.994350	-0.630054	-0.223369
O	0.680794	1.504864	0.059412
O	1.371065	-0.925257	1.033734
Se	0.972261	-0.060252	-0.277313

SeO_2···CH_3CHS

Atom	*a* (Å)	*b* (Å)	*c* (Å)
C	1.948736	0.348730	0.049809
H	1.226006	1.172801	0.055218
C	3.358450	0.690203	0.372564
H	4.010554	-0.177704	0.340628
H	3.711649	1.449625	-0.328719
H	3.393229	1.145784	1.365029
O	-0.778634	1.516905	-0.267423
O	-1.742576	-0.477101	1.286255
Se	-1.363117	-0.001494	-0.215797
S	1.395695	-1.130734	-0.298746

SeO_2···CH_3CSCH_3

Atom	*a* (Å)	*b* (Å)	*c* (Å)
C	2.075247	0.150792	-0.111193
C	3.503494	0.235037	0.328114
H	3.888441	-0.728991	0.646030
H	4.109373	0.619396	-0.496037
H	3.587651	0.957395	1.143916
C	1.429444	1.455439	-0.448204
H	1.157243	1.956510	0.485971
H	2.138553	2.100035	-0.969488
H	0.528567	1.336146	-1.042261
O	-1.178930	0.798372	1.320272
O	-1.827920	0.532365	-1.291067
Se	-1.591824	-0.219848	0.126409

Se	-1.386965	-0.310596	-0.006299	S	1.294867	-1.278698	-0.181999
SeO_2···HCOOH				SeO_2···HCSOH			
Atom	*a* (Å)	*b* (Å)	*c* (Å)	Atom	*a* (Å)	*b* (Å)	*c* (Å)
O	-1.493695	-0.731022	1.093357	O	-1.794973	-0.549883	1.103132
O	-0.289422	1.420363	0.000615	O	-0.525978	1.481231	-0.114647
Se	-1.017243	-0.007301	-0.269061	Se	-1.134617	-0.031426	-0.273938
C	2.353176	-0.478942	-0.004865	C	2.349389	0.117669	0.172761
O	1.382080	-1.107404	-0.393370	O	1.989626	1.359297	0.268528
O	2.359694	0.780082	0.399345	H	3.417881	-0.014533	0.329988
H	3.352727	-0.914884	0.049411	H	0.989916	1.465250	0.120473
H	1.445241	1.140619	0.328288	S	1.420216	-1.213337	-0.139327
